# The effect of wearing sanitary napkins of different thicknesses on physiological and psychological responses in Muslim females

**DOI:** 10.1186/1880-6805-33-28

**Published:** 2014-09-04

**Authors:** Nazirah G Mohamed, Nurdiana Z Abidin, Kim S Law, Mika Abe, Megumi Suzuki, Ahmad M Che Muhamed, Rabindarjeet Singh

**Affiliations:** 1 Lifestyle Science Cluster, Advanced Medical and Dental Institute, Universiti Sains Malaysia, Bertam, 13200 Kepala Batas, Penang, Malaysia; 2 R&D, Kao Corporation, 2606 Akabane, Ichikai-machi, Haga-gun, Tochigi 321-3497, Japan

**Keywords:** Comfort, Menstruation, Physical activity, Sanitary napkin

## Abstract

**Background:**

Menstruation is associated with significant unpleasantness, and wearing a sanitary napkin (SN) during menses causes discomfort. In addition, many Muslim women use a thick type of SN during menses due to the religious requirement that even disposable SNs be washed before disposal. Therefore, the objective of this study was to measure the physiological and psychological responses to wearing SNs of different thicknesses during menstruation and non-menstruation phases at rest and during physical activity/exercise among Muslim women.

**Methods:**

Eighteen Muslim females were randomly assigned to wear an ultra slim type (US, thin) or a maxi type (MT, thick) SN on two different occasions (i.e., during non-menses and menses). Each subject tested both types of SN. Upon arriving at the laboratory, each subject was equipped with an ambulatory electrocardiograph and rested in a seated position for 10 min. She was then given either an US or MT SN, put it in place, and rested in a seated position for 10 min. Each subject then walked at 3 km/h for 10 min, sat resting for 10 min, and then walked at 5 km/h for another 10 min. At the end of each 10-min stage, subjects marked their feelings of discomfort on the visual analog scale (VAS). Perceived exertion during exercise was evaluated using the Borg scale. Heart rate and low frequency-to-high frequency ratio (LF/HF) of heart rate variability were continuously recorded during rest and exercise.

**Results:**

During both the non-menses and menses trials, VAS and LF/HF were significantly lower in subjects using the US SN compared to the MT SN. These results indicate that when wearing the US SN, subjects were more comfortable and did not increase sympathetic activities. Meanwhile, perceived exertion during exercise had no significant difference between US and MT although the means of the scores for US tended to be lower than those of MT.

**Conclusions:**

The results of this study (VAS and LF/HF) indicate that wearing an US SN induces less physiological and psychological stress compared to wearing a MT SN. Thus, use of the former will empower women to live their lives with vitality during menses.

## Background

Female menstruation is associated with significant unpleasant symptoms, which can be physical, behavioral, and emotional [1,2]. Consequently, reducing mental stress during the menstruation period is an important quality of life issue for women. Wearing a sanitary napkin (SN) is believed to influence mental stress responses of women during daily living activities [1,2]. Although mental stress is a psychological response, it affects several physiological processes in the human body. Generally, the human sensory receptors respond to physical stimuli, such as touch and pain, and trigger physiological changes in the body as interpreted by the autonomic nervous system [3]. The parasympathetic nervous system is suppressed and the sympathetic nervous system (SNS) is activated [3]. This causes physiological secretion of epinephrine and norepinephrine, which leads to vasoconstriction of blood vessels, increased muscle tension, and changes in heart rate and heart rate variability (HRV) [3].

In the past, researchers have used HRV to measure mental stress [1-5]. In particular, the high frequency (HF) band (0.15 to 0.4 Hz) in frequency-domain analysis has been regarded as the marker of parasympathetic (vagal) activity, and the low frequency (LF) band (0.04 to 0.15 Hz) has been regarded as the marker of sympathovagal interaction, especially sympathetic activity. Consequently, the LF-to-HF (LF/HF) ratio represents the sympathovagal balance [6,7]. HRV in response to stress differs between genders, and women’s neuroendocrine responses are often unpredictable [3].

Previous studies have used different types of SNs as the physical stimuli and measured physiological responses to their use in order to find ways to improve the quality of life of women during menses [1,4,5]. For example, Park and Watanuki [1,2] reported that, although the use of SNs increased physiological loading, their use did not result in significant effects on the LF and HF components or the LF/HF ratio of HRV at 20, 30, and 40 min after application compared to during the 10 min resting state. In a laboratory-based experiment, application of different types of SN had different physiological and psychological outcomes [1,2]. Mechanical stimulation by SNs with higher roughness (frictional coefficient = 0.312) increased SNS activities and brain arousal compared to SNs with lower roughness (frictional coefficient = 0.142) [1]. Researchers who focused on physiological changes caused by application of SNs of different thicknesses over the menstrual cycle reported that women felt more comfortable using thin type SNs compared to thick type SNs [4,5]. Thin type SNs caused less mechanical stimulation and did not increase the SNS activities compared to the thick type SN [4,5].

Muslim females prefer to use the thick type SN due to cultural and religious practices, as SNs must be washed before disposal. This is based on tradition passed down for generations as in the older days there were no napkins and many used old cloths as napkins [8]. In addition, many believe that if used SNs are not washed before being thrown away, one risks being afflicted with hysteria and disturbances and is unhygienic. Research on the psychological and physiological changes that occur in women when wearing SNs of different thicknesses is limited and, to our knowledge, there have been no studies on the effect of wearing SNs during exercise/physical activity among Muslim women. Therefore, the goal of this study was to measure the physiological and psychological responses to wearing SNs of different thicknesses during menstruation and non-menstruation phases at rest and during physical activity/exercise among Muslim women.

## Methods

### Subjects

Unmarried Muslim females were solicited from the Institute to participate in this study. Eighteen unmarried Muslim females with regular menstrual cycles (cycle range 21 to 39 days) volunteered to participated in the study during the non-menses period (Experiment I: age 24.6 ± 1.4 years, height 155.3 ± 4.5 cm, weight 51.2 ± 12.4 kg, BMI 21.2 ± 4.8), and 18 unmarried Muslim females participated during their menses period (Experiment II: age 25.0 ± 1.4 years, height 156.2 ± 5.0 cm, weight 51.8 ± 9.9 kg, BMI 21.1 ± 3.7), 16 of whom had participated in Experiment I. None of the subjects had any premenstrual syndrome, which was assessed via a questionnaire before participation in the study. None of the subjects had any menstrual pains during their menses or took any analgesics to control menstrual pains. Written informed consent was obtained from all subjects after a full explanation of the study purpose and protocol. Subjects were asked to abstain from eating, smoking, and exercise at least 1 h before the experiments. This study was approved by the Human Ethics Committee of Universiti Sains Malaysia.

### Experimental conditions

To avoid potential diurnal variations, subjects were always tested at the same time of day (between 13:30 h and 17:00 h) in the same quiet, temperature-controlled room (26.3 ± 1.9°C, 51.6 ± 8.7% relative humidity).

### Materials

Two different types of SN were used in this study: ultra slim (US, thin; Laurier Perfect Comfort Ultra Slim) and maxi type (MT, thick; Laurier Active Comfort Super Maxi) (Table 1). Both types of SN were marketed products (Kao Corporation) consisting of three layers: top-sheet (non-woven sheet), absorbent sheet (MT: Pulp; US: pulp + specially designed washable absorbing polymers), and back sheet (polythene film).

**Table 1:** Description of sanitary napkins used in the study

	**Thickness**	**Length**	**Wing**
**Maxi type (MT)**	9 mm	22 cm	YES
**Ultra slim (US)**	4 mm	22 cm	YES

### Experimental design

A crossover repeated-measures design with random assignment was used. Subjects were required to complete two phases of the experiment: Experiment I was the non-menses phase and Experiment II was the menses phase. The menses phase represented a more complex situation than the non-menses phase because of menstrual pain, worries about leakage, and so on. Thus, to evaluate the effect of SNs on the subject’s comfort, experiments were executed during the non-menses phase. For each experiment, subjects had to complete two visits, one using the US SN and the other using the MT SN (assigned in a randomized order). For Experiment I, subjects completed the first and second visits on two continuous days, whereas for Experiment II the visits took place in two different months, with the gap between the first and second visit being no more than 3 months.

### Procedures

**Figure 1:**
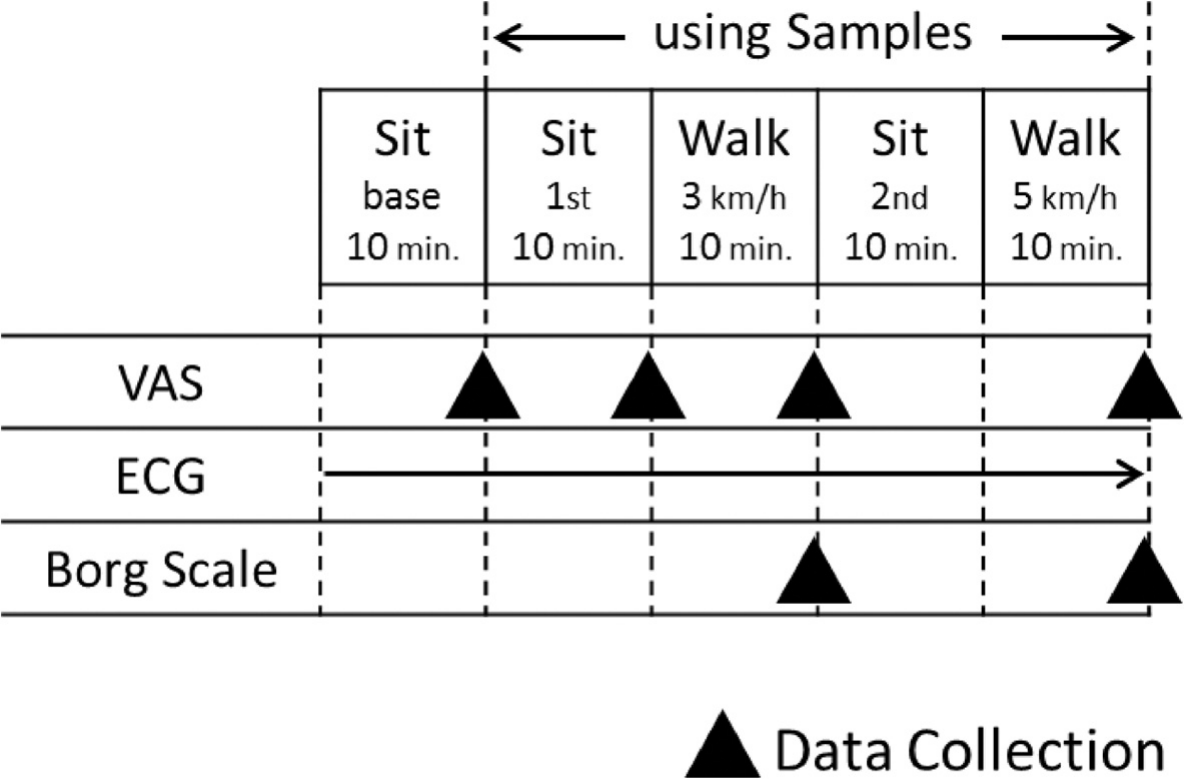
**Flow of the project design.** VAS = visual analog scale for determination of feeling of discomfort; ECG = electrocardiogram, which was continuously monitored.

Upon arrival in the laboratory for Experiment I, subjects were required to fill out a pre-questionnaire to identify the type of SN used regularly during their heavy flow menses days. Subjects were then equipped with an ambulatory electrocardiogram monitor (Active Tracer, Model: AC-301A, Japan) with electrodes placed on three sites on the chest, and they rested in a seated position for 10 min. Subjects were then given either an US or MT SN (given in a randomized order, and subject were blinded to the type of SN given), they were asked to put it in place, and they again rested in a seated position for 10 min. Subjects then walked at 3 km/h on a treadmill for 10 min, sat resting for 10 min, and then continued walking briskly at 5 km/h on a treadmill for another 10 min (Figure 1). At the end of each 10-min stage, subjects were asked to mark their feelings of discomfort on a visual analog scale (VAS) as described by Scott and Huskisson [9]. For the treadmill stage, immediately upon completing the 10 min of walking, subjects were required to point immediately on the printed 10-point Borg scale to record their level of perceived exertion [10]. HRV was recorded continuously during rest and exercise by the electrocardiogram monitor, and the frequency of the R-R interval was analyzed by the MemCalc method using MemCalc/Tarawa software. From the analysis, the LF/HF ratio was calculated. The data collected during minutes 5 to 8 of each stage were used for the analysis. On the second visit (the following day), subjects repeated the same protocols while wearing the other SN type.

Upon arrival at the laboratory for Experiment II, subjects were given an US SN, asked to put it in place, and rested in the seated position for 10 min. They then were given either an US or MT SN in randomized order and asked to put it in place. The subjects completed this experiment during the second and third days after the onset of menstruation, and the average value of both days was used in the data analysis. If a subject was randomly assigned to use the MT SN on the first menses visit (second and third day of menstruation), she was given the US SN on her second menses visit (second and third day of menstruation), and vice versa. At the end of Experiment II, subjects were required to fill out the post-questionnaire to identify the type of SN that made them feel more active.

### Statistical analysis

Statistical analysis was performed using SPSS software (version 22, IBM, USA). The data of VAS, LF/HF, and Borg scale were evaluated using a two-way (sample × stage) repeated measures analysis of variance (ANOVA) with Bonferroni *post hoc* test to identify the overall difference between US and MT SNs. The levels of significance were set at *P* <0.05. Data are presented as relative values to eliminate the effect of individual and daily differences.

## Results

**Figure 2:**
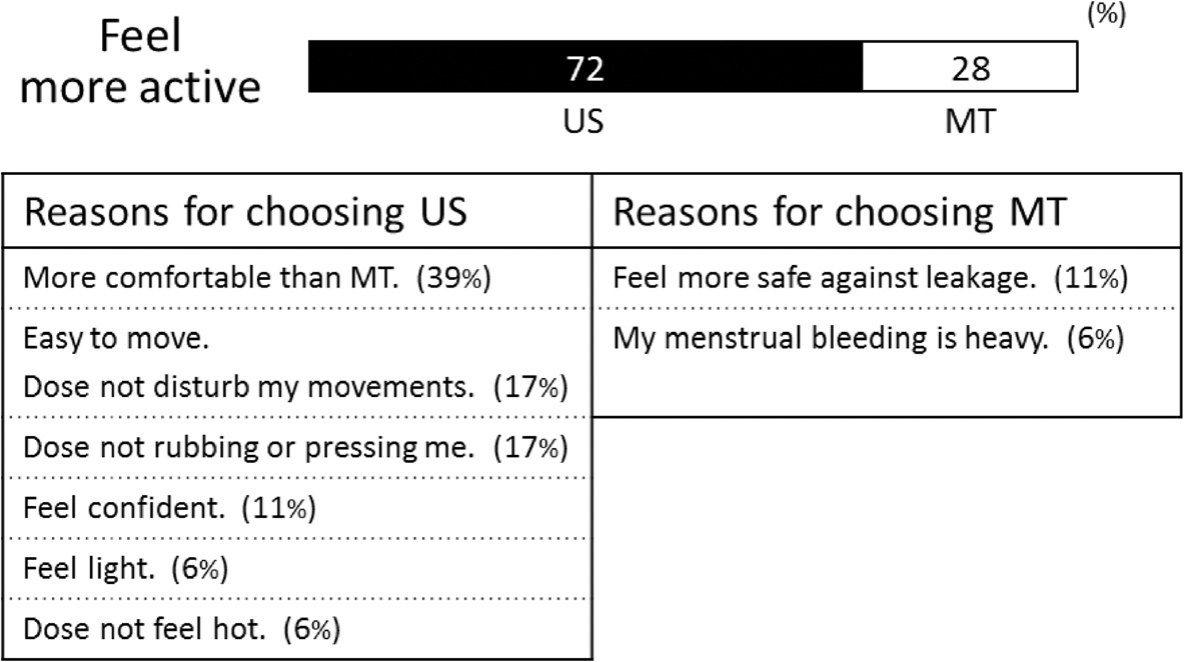
**Analysis of the questionnaire on the usage of different type of sanitary napkins.** US = ultra slim type of sanitary napkin; MT = maxi type of sanitary napkin.

Analyses of the pre-questionnaire showed that 89% of the subjects who participated in Experiment I (non-menses phase) and 94% who participated in Experiment II (menses phase) used a thick type SN during their heavy flow menstruation days. The post-questionnaire revealed that 72% of the subjects in Experiment II felt more active wearing the US SN compared to the MT SN. The post-questionnaire answers also revealed the subjects’ reasons for choosing their preferred type of SN (Figure 2).

**Figure 3:**
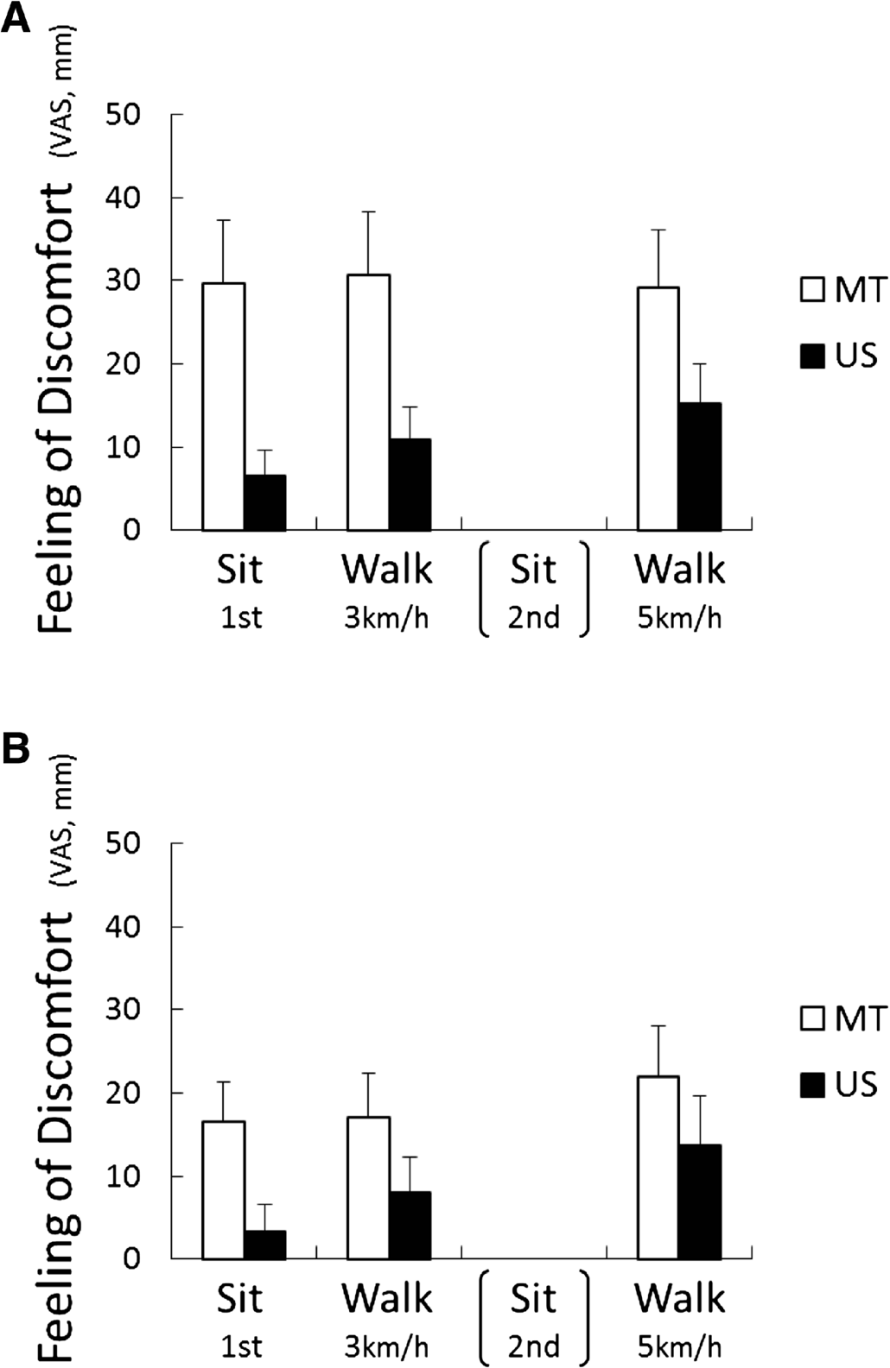
**Relative values of feeling of discomfort as measured using VAS during the non-menses (A) and menses (B) phases of the experiment.** US = ultra slim type of sanitary napkin; MT = maxi type of sanitary napkin. The main effect of “sample” was significant during the non-menses phase (*F*_(1, 34)_ = 15.44, *P* <0.001) and the menses phase (*F*_(1, 34)_ = 6.60, *P* <0.05) by a two-way (sample × stage) ANOVA.

There was a consistent trend of reduced feelings of discomfort during seated rest and exercise when wearing the US SN compared to the MT SN in both experiments. The main effect of “sample” was significant (Figure 3; *F*_(1, 34)_ = 15.44, *P* <0.001 during the non-menses phase and *F*_(1, 34)_ = 6.60, *P* <0.05 during the menses phase) and VAS values were significantly reduced in the subjects wearing the US SN compared to the MT SN (Experiment I: *P* <0.001, Experiment II: *P* <0.05).

**Figure 4:**
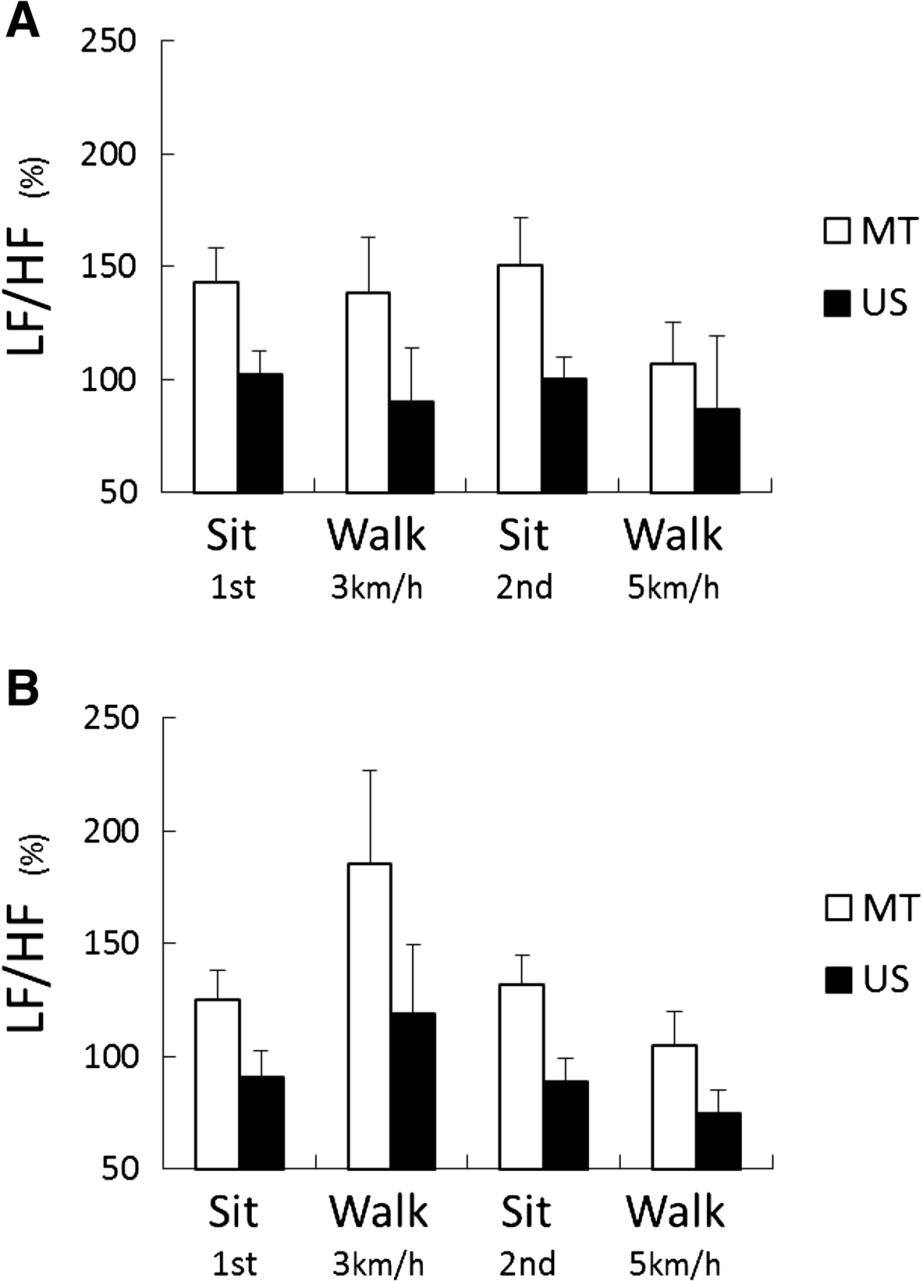
**Relative values of LF/HF during the non-menses (A) and menses (B) phases of the experiment.** US = ultra slim type of sanitary napkin; MT = maxi type of sanitary napkin. The main effect of “sample” was significant during the non-menses phase (*F*_(1, 34)_ = 7.30, *P* <0.01) and the menses phase (*F*_(1, 34)_ = 8.55, *P* <0.01), and the main effect of “stage” was significant during the menses phase only (*F*_(3, 102)_ = 3.17, *P* <0.05) by a two-way (sample × stage) ANOVA.

The LF/HF ratio was lower in subjects wearing the US SN compared to the MT SN in all stages of the experiments, and the significant main effect of “sample” was identified (Figure 4; *F*_(1, 34)_ = 7.30, *P* <0.01 during the non-menses phase and *F*_(1, 34)_ = 8.55, *P* <0.01 during the menses phase). The main effect of “stage” was significant in the menses phase only (*F*_(3, 102)_ = 3.17, *P* <0.05). No interaction between “sample” and “stage” was observed. The LF/HF ratio for the US SN was significantly lower than that for MT in both experiments (Experiments I and II: *P* <0.01).

**Figure 5:**
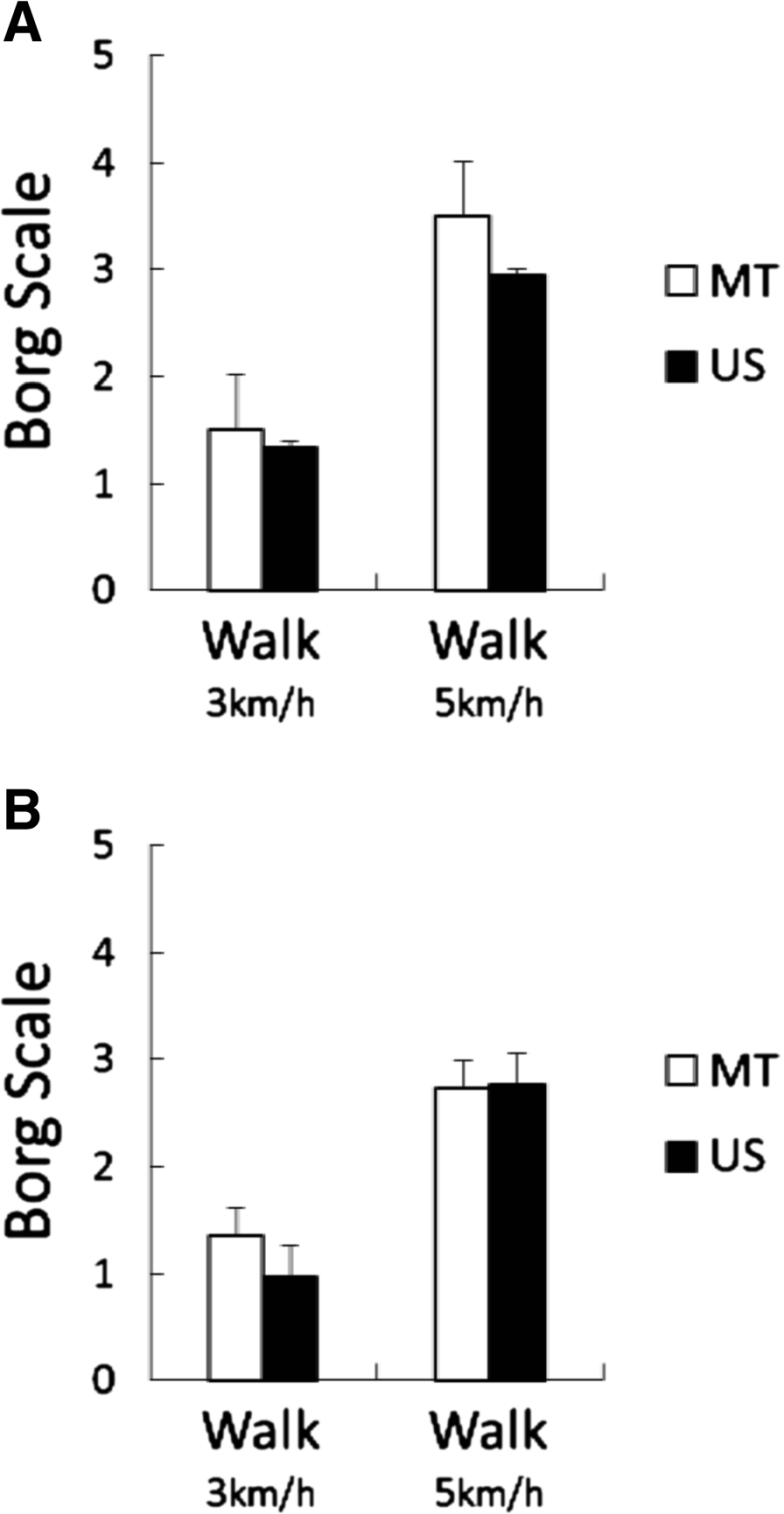
**Results of Borg scale during the non-menses phase (A) and the menses phase (B) of the experiment.** US = ultra slim type of sanitary napkin; MT = maxi type of sanitary napkin. The main effect of “stage” was significant during the non-menses phase (*F*_(1, 34)_ = 19.19, *P* <0.001) and the menses phase (*F*_(1, 32)_ = 43.75, *P* <0.001) by a two-way (sample × stage) ANOVA.

The means of scores on the Borg scale (Figure 5) were lower when wearing the US SN compared to the MT SN at the end of the 3 km/h walk phase in Experiments I and II, and the 5 km/h walk phase in Experiment I. However, there was no significance in the main effect of “sample”. The main effect of “stage” was significant in both experiments (*F*_(1, 34)_ = 19.19, *P* <0.001 during the non-menses phase and *F*_(1, 32)_ = 43.75, *P* <0.001 during the menses phase).

## Discussion

During menses, Malaysian Muslim women generally prefer to use a thick type SN without absorbing polymers to increase their confidence level, to be free from worries about leakage, and to enable easy washing of used SNs, which is required for cultural and religious reasons. In the current study, the majority of the Malaysian Muslim females (90%) reported using a thick type SN during their menstruation period. In contrast, another study reported that only 38% of Japanese women use a thick type SN during their heavy flow menstruation days [5].

As was found in the Japanese study [4,5], wearing SNs of different thicknesses elucidated different physiological and psychological responses in Malaysian women. Compared to subjects wearing the MT SN, those wearing the US SN showed decreased percentage changes of the LF/HF ratio and lower relative VAS scores. Thus, wearing the US SN did not increase SNS activities, and the subjects using this type of SN felt more comfortable (Figures 3 and 4). These results indicate that wearing the US SN reduced physiological and psychological stress in the subjects, especially during menstruation, even though they had previously been used to using thick type SNs.

Regarding perceived exertion (as measured using the Borg scale), a previous study reported that a feeling of comfort and the Borg scale exhibited an inverse relationship [11]. In our study, although there were no significant differences between US and MT, the means of scores tended to be lower when wearing the US SN compared to the MT SN (Figure 5). In addition, 72% of the subjects reported feeling more active when wearing the US SN compared to the MT SN in the post-questionnaire (Figure 2). Feeling more comfortable when wearing the US SN might have caused the subjects to feel more active or experience less physical fatigue [11-13]. However, the connection between the use of the different SN types and physical activity remains unclear. Hence, future studies should objectively measure whether wearing an US SN decreases physical fatigue or improves exercise performance; the answer will better explain the relationship between US SNs and physical activity. Furthermore, it appears that the use of US SNs will allow women to be more active and manage their stress during menstruation, thereby increasing their quality of life.

### Study limitations

One of the limitations of this study was that serum levels of ovarian hormones (estradiol and progesterone) and thyroid hormones were not measured. Concentrations of these hormones change during different phases of the menstrual cycle and differ among subjects. Further, they can modulate HRV and the related autonomic nervous system. Additionally, the subjects were assumed to be in good mental and physical health, but they did not undergo a medical examination. These factors should be taken into account in future studies.

## Conclusions

The physiological and psychological differences (LF/HF ratio and VAS) between subjects wearing the US SN and the MT SN exhibited a consistent trend in both Experiments I and II (i.e., the ratio was lower in those using the US SN). This finding illustrates that the US SN is more comfortable to wear than the MT SN, and comfort is an important factor that could eliminate physiological and psychological stress and unpleasant symptoms during menstruation.

## Abbreviations

ANOVA: Analysis of variance; HF: High frequency; HRV: Heart rate variability; LF: Low frequency; MT: Maxi type; SN: Sanitary napkin; SNS: Sympathetic nervous system; US: Ultra slim; VAS: Visual analog scale.

## Competing interests

The authors declare that they have no competing interests.

## Authors’ contributions

RS, MA, and AMCM designed the study. NGM, NZA, and LKS conducted the experimental work. NGM, MA, MS, and RS analyzed data, prepared figures, and drafted the manuscript. All authors participated in data interpretation and revised the manuscript. The final version of manuscript was approved by all authors.
